# Prediction of hemodynamic tolerance of intermittent hemodialysis in critically ill patients: a cohort study

**DOI:** 10.1038/s41598-021-03110-4

**Published:** 2021-12-08

**Authors:** Rogerio da Hora Passos, Juliana Ribeiro Caldas, Joao Gabriel Rosa Ramos, Erica Batista dos Santos Galvão de Melo, Marcelo Augusto Duarte Silveira, Paulo Benigno Pena Batista

**Affiliations:** 1 Critical Care Unit Hospital São Rafael, Salvador, Brazil; 2 Instituto de Pesquisa e Ensino D’OR (IDOR), Salvador, Brazil; 3 Universidade de Salvador- UNIFACS, Salvador, Brazil; 4 Escola Bahiana de Medicina e Saúde Pública- EBMSP, Salvador, Brazil

**Keywords:** Haemodialysis, Radiography

## Abstract

The evaluation and management of fluid balance are key challenges when caring for critically ill patients requiring renal replacement therapy. The aim of this study was to assess the ability of clinical judgment and other variables to predict the occurrence of hypotension during intermittent hemodialysis (IHD) in critically ill patients. This was a prospective, observational, single-center study involving critically ill patients undergoing IHD. The clinical judgment of hypervolemia was determined by the managing nephrologists and critical care physicians in charge of the patients on the basis of the clinical data used to calculate the ultrafiltration volume and rate for each dialysis treatment. Seventy-nine (31.9%) patients presented with hypotension during IHD. Patients were perceived as being hypervolemic in 109 (43.9%) of the cases by nephrologists and in 107 (43.1%) by intensivists. The agreement between nephrologists and intensivists was weak (kappa = 0.561). Receiver operating characteristic curve analysis yielded an AUC of 0.81 (95% CI 0.75 to 0.84; *P* < 0.0001), and a cutoff value of 70 mm for the vascular pedicle width (VPW) had the highest accuracy for the prediction of the absence of hypotension. The clinical judgment of hypervolemia did not predict hypotension during IHD. The high predictive ability of the VPW may assist clinicians with critical thinking.

The evaluation and management of fluid balance are key challenges when caring for critically ill patients requiring renal replacement therapy (RRT)^1,2^. Despite considerable advances in the assessment of dialysis adequacy with respect to solute removal and data suggesting that net ultrafiltration may be associated with the outcomes^3–5^, there is currently no specific measure of adequacy for fluid removal^6,7^. Intradialytic hypotension is a common complication associated with RRT; it may be associated with the ultrafiltration rate and can cause further ischemic injury to the recovering kidneys, thereby potentially reducing the probability of renal recovery^4,8^. Therefore, the selection of the optimal ultrafiltration rate that will not result in any adverse clinical consequences depends on an accurate estimation of the patient’s fluid status and hemodynamics, an adequate understanding of the principles of fluid overload treatment with ultrafiltration, and clear treatment goals^2,9,10^, although there are several methods to evaluated volume responsiveness in critically ill patients, these tools are usually used to evaluate patients in a life-threatening condition, as circulatory shock, not to predict hypotension during RRT.

The most popular approach adopted in the management of fluid overload is to use a clinician-set ultrafiltration target. There have been no well-conducted studies showing that any strategy for blood volume assessment is associated with a reduced risk of hypotension during intermittent hemodialysis (IHD)^11^. Usually, the nephrologist and critical care physician in charge of acute kidney injury (AKI) patients requiring hemodialysis have relied on their subjective clinical judgment, based on clinical data such as the physical examination, hemodynamic monitoring, perfusion parameters and radiological features, to calculate the ultrafiltration volume and rate for each dialysis treatment^6,9,11,12^. The aim of this study was to assess the ability of clinical judgment and other variables to predict the occurrence of hypotension during IHD in critically ill patients.

## Methods

A prospective, observational, single-center study was performed between January 2015 and April 2018 in a 30-bed medical intensive care unit in a tertiary hospital in Brazil. After ethics approval (CAAE: 89428318.000005029), we included consenting critically ill patients if they fulfilled the following criteria: age > 18 years old with AKI defined by Kidney Disease: Improving Global Outcomes (KDIGO) 3 in need of IHD, patients with chronic kidney disease were excluded. Patients in which the components of the silhouette have been altered by vascular or other mediastinal disease that might influence VPW measurements and artfactually alter its predictive value were also excluded.

IHD sessions were managed by technical nurses, and the indication for and settings of IHD sessions were the responsibility of the nephrology team in charge of the patient. RRT was initiated on the basis of the standard clinical guidelines, including AKI with hemodynamic stability, ongoing hypercatabolism, hyperkalemia, severe metabolic acidosis, presumed volume overload, respiratory distress or some combination of these factors. The indication for IHD rather than continuous dialysis procedures in our unit is based on the absence of vasopressors or use of a low dose of vasopressors (norepinephrine dose ≤ 0.3 mg kg^−1^ min^−1^) for at least 6 h before the initiation of dialysis with the maintenance of a mean arterial pressure (MAP) ≥ 65 mmHg. For each patient, a combination of clinical, laboratory, and static and dynamic hemodynamic variables was used to assess the volume status and inform clinical decision making regarding the ultrafiltration rate. IHD was performed with Fresenius 4008 S hemodialysis generators and dialysate concentrate solutions with a 1.75 mmol/L calcium concentration. The IHD settings regarding the blood and dialysate flow rates, dialysate temperature and dialysate sodium concentration were prescribed by the physician in charge.

Hypotension was defined as the first occurrence of a MAP less than 65 mmHg during the session^13,14^.

All methods were performed in accordance with the relevant guidelines and regulations.

### Clinical judgment of hypervolemia

Hypervolemia was determined by the managing nephrologists and critical care physicians in charge of the cases using a systematic clinical approach including patient history, symptoms, physical examination including peripheral edema, laboratory parameters, ventilator settings for those on mechanical ventilation and routine diagnostic techniques, as available.

The measurement of the vascular pedicle width (VPW) was performed with chest X ray (CXR). The right border of the VPW was the point at which the superior vena cava crossed the right main bronchus. The left border was the point at which the subclavian artery exited the aorta. The VPW was defined as the horizontal distance between those two points. The cardiothoracic ratio (CTR) was evaluated by the Danzer method^15^. All CXRs were analyzed three times by two independent observers. The inter- and intraobserver coefficients of variation were 2.9 and 6.1%, respectively.

Data were analyzed using the Statistical Package for Social Sciences (SPSS Inc., Chicago, IL, USA). The Kolmogorov–Smirnov test was used, and histograms and normal quantile plots were examined to verify the normality of the distribution of continuous variables. Difference testing between groups was performed using two-tailed *t* tests, Mann–Whitney *U* tests, chi-square tests, and Fisher’s exact tests, as appropriate. To evaluate the influence of baseline characteristics on hypotension in AKI patients requiring IHD, we performed a multivariable logistic regression. All tests were two-tailed, and a *P *value less than 0.001 was considered significant in the identification of the associated independent variables. Receiver operating characteristic (ROC) curves were constructed using different cutoffs of the VPW and CTR to identify the sensitivities and specificities for each value, and they were also calculated for the physician perception of fluid status and presence of peripheral edema. Agreement between nephrologists and intensivists was evaluated by the kappa coefficient.

### Ethics approval and consent to participate

The study was approved and the need to obtain written informed consent was waived for this observational and noninterventional study by the Ethics Committee of Centro de Estudos Egaz Muniz (CAAE: 89428318.000005029).

## Results

Two hundred forty-eight AKI patients requiring IHD were included in the study. Demographic data and patient characteristics at the onset of IHD are reported in Table 1. Seventy-nine (31.9%) patients presented with hypotension during an IHD session.

**Table 1:** Distribution of parameters according to the occurrence of hypotension (univariate analysis).

Variable	Total cohort	Hypotension		*p*
		No	Yes	
	N = 248	169 (68.1%)	79 (31.9%)	
**Sex**				0.684
Male, n (%)	149 (60.1%)	103 (60.9%)	46 (58.2%)	
Female, n (%)	99 (39.9%)	66 (39.0%)	33 (41.8%)	
Age (years)	68.0 (58.2–76)	66 (55–76)	70 (64–76)	0.018
Charlson comorbidity index score	10 (8–12)	10 (8–12)	10 (8–12)	0.116
APACHE II	15 (12–18)	14 (12–18)	16 (13–18)	0.215
SOFA score	8 (6–100	8 (6–10)	8 (7–10)	0.057
Sepsis, n (%)	123 (49.6%)	71 (42%)	52 (65.8%)	< 0.001
Use of norepinephrine, n (%)	37 (14.9%)	5 (3%)	32 (40.5%)	< 0.001
Mechanical ventilation, n (%)	34 (13.7%)	14 (8.3%)	20 (25.3%)	< 0.001
Cumulative Fluid balance (milliliters)	1772 (1540–2320)	1800 (1535–2320)	1750 (1540–2350)	0.762
**Indicators of hypervolemia**				
Nephrologist	109 (43.9%)	92 (54.4%)	17 (21.5%)	< 0.001
Intensivist	107 (43.1%)	80 (47.3%)	18 (22.8%)	< 0.001
Peripheral edema	119 (48.0%)	101 (59.8%)	18 (22.8%)	< 0.001
Cardiothoracic index	52 (50–54)	52 (50–54)	54 (52–56)	0.003
Cardiothoracic index < 0.55	189 (76.2%)	136 (80.5%)	53 (67.1%)	0.025
VPW	68 (66–72)	72 (68–74)	65 (64–68)	< 0.001
VPW < 70	143 (57.7%)	76 (45.0%)	68 (86.1%)	< 0.001
**Dialysis data**				
Duration (min)	240 (180–240)	240 (180–240)	210 (150–240)	0.140
Ultrafiltration (milliliters)	1000 (200–2000)	1.242.01	893.03	0.020
- Blood flow rate (milliliters/min)	300 (250–300)			0.010
200		24 (14.2%)	24 (30.4%)	
250		47 (27.8%)	16 (20.3%)	
300		98 (58%)	38 (48.1%)	
350		0 (0%)	1 (1.3%)	
-Dialysate flow rate (milliliters/min)	500 (500–500)			0.002
300		12 (7.1%)	17 (21.5%)	
320		0 (0%)	1 (1.3%)	
500		157 (92.9%)	61 (77.2%)	
Temperature (^o^C)	36 (36–36)	36.0 (35.5–36.5)	36.0 (35.5–36.5)	0.416
Sodium (mEq/L)	138 (138–140)	138 (138–140)	138 (135–141)	
**Systemic data**				
Systolic blood pressure (mmHg)	132 (114.2–152)	140 (123–160)	114 (103–135)	< 0.001
Diastolic blood pressure (mmHg)	70 (60.2–80.7)	71 (64–86)	64 (58–73)	< 0.001
Mean blood pressure (mmHg)	89.5 (79.0–105.0)	94 (84–109)	81 (75–90)	< 0.001
**- Blood test data**				
Hemoglobin (g/dL)	9 (7.6–10.1)	9 (7.4–10.1)	9 (7.8–10.2)	0.839
Bicarbonate (mEq/L)	20 (18–23)	20 (18–23)	20 (16–22)	0.321
Sodium (mEq/L		138 (135–141)	139 (134–141)	0.565
Urea (mg/dL)	143 (114.2–194)	143 (110–195)	142 (119–194)	0.659
Lactate (mmol/l)	1.4 (1.1–1.9)	1.3 (1.1–1.7)	1.7 (1.2–2.2)	< 0.001

*SOFA* sequential organ failure assessment, *APACHE* acute physiology and chronic health evaluation, *CTR* cardiothoracic index.

The IHD settings were different between hypotensive and nonhypotensive sessions. In general, the amount of ultrafiltration, blood flow rate and dialysate flow rate were lower in hypotensive sessions than in nonhypotensive sessions **(**Table 1). The diagnosis of sepsis, the use of norepinephrine, a lower MAP, a higher lactate level, the size of the VPW, the presence of peripheral edema and the need for mechanical ventilation were associated with the development of hypotension during IHD (Table 1). However, in multiple logistic regression, only the need for norepinephrine [OR (95% CI) = 16 (4–65)] and the size of the VPW [(OR 95% CI) = 0.73 (0.65–0.82)] were independently associated with the development of hypotension during an IHD session (Table 2).

**Table 2:** Multivariate logistic regression of the variables significant in univariate analysis in the patients with hypotension.

Variable	Parameter estimate	Standard error	*Odds ratio*	95% CI	P
Age	− 0.001	0.014	0.999	0.971–1.027	0.925
Blood flow rate	− 0.001	0.006	0.999	0.988–1.010	0.858
Dialysate flow rate	− 0.007	0.004	0.993	0.985–1.000	0.043
Ultrafiltration	0.001	0.001	1.111	0.951–1.001	0.086
Mean blood pressure	− 0.025	0.013	0.976	0.934–0.991	0.061
Lactate	− 0.121	0.297	0.886	0.495–1.585	0.684
Use of norepinephrine	2.796	0.706	16.381	4.109–65.304	< 0.001
Peripheral edema	− 0.828	0.413	0.437	0.194–0.082	0.045
VPW	− 0.312	0.058	0.732	0.653–0.821	< 0.001
Cardiothoracic index	0.105	0.061	0.437	0.194–0.982	0.086
Mechanical ventilation	− 0.323	0.686	0.724	0.189–2.777	0.276
Sepsis	0.293	0.706	1.341	0.591–3.040	0.925
Constant	20.815				

*VPW* vascular pedicle width.

Patients were perceived as hypervolemic in 109 (43.9%) of the cases by nephrologists and in 107 (43.1%) by intensivists. Nevertheless, the specificity and sensitivity for the prediction of hypotension were 45.6% and 21.5% for nephrologists and 15.6% and 55.4% for intensivists, respectively. The agreement between nephrologists and intensivists was weak (kappa = 0561). A VPW cutoff > 70 mm yielded a sensitivity of 86.1% (95% CI 70.3 to 92.3) and a specificity of 55% (95% CI 43.2 to 73.2). However, the positive predictive value was only 47.2% (95% CI 33.2 to 59.3%), and the negative predictive value was 89.4% (95% CI 74.2 to 94.5%) (Table 3).

**Table 3:** Sensibility, specificity, and positive and negative predictive values of the variables for the detection of hypotension.

	Specificity	Sensibility	PPV	NPV
VPW	55%	86.1%	47.2%	89.4%
Peripheral edema	59.8%	77.2%	47.3%	84.9%
Nephrologist perception	45.6%	21.5%	15.6%	55.4%
Intensivist perception	47.3%	22.8%	16.8%	56.7%
CTR	19.5%	67.1%	28.0%	55.9%

*VPW* vascular pedicle width, *CTR* cardiothoracic index, *PPV* positive predictive value, *NPV* negative predictive value.

**Figure 1:**
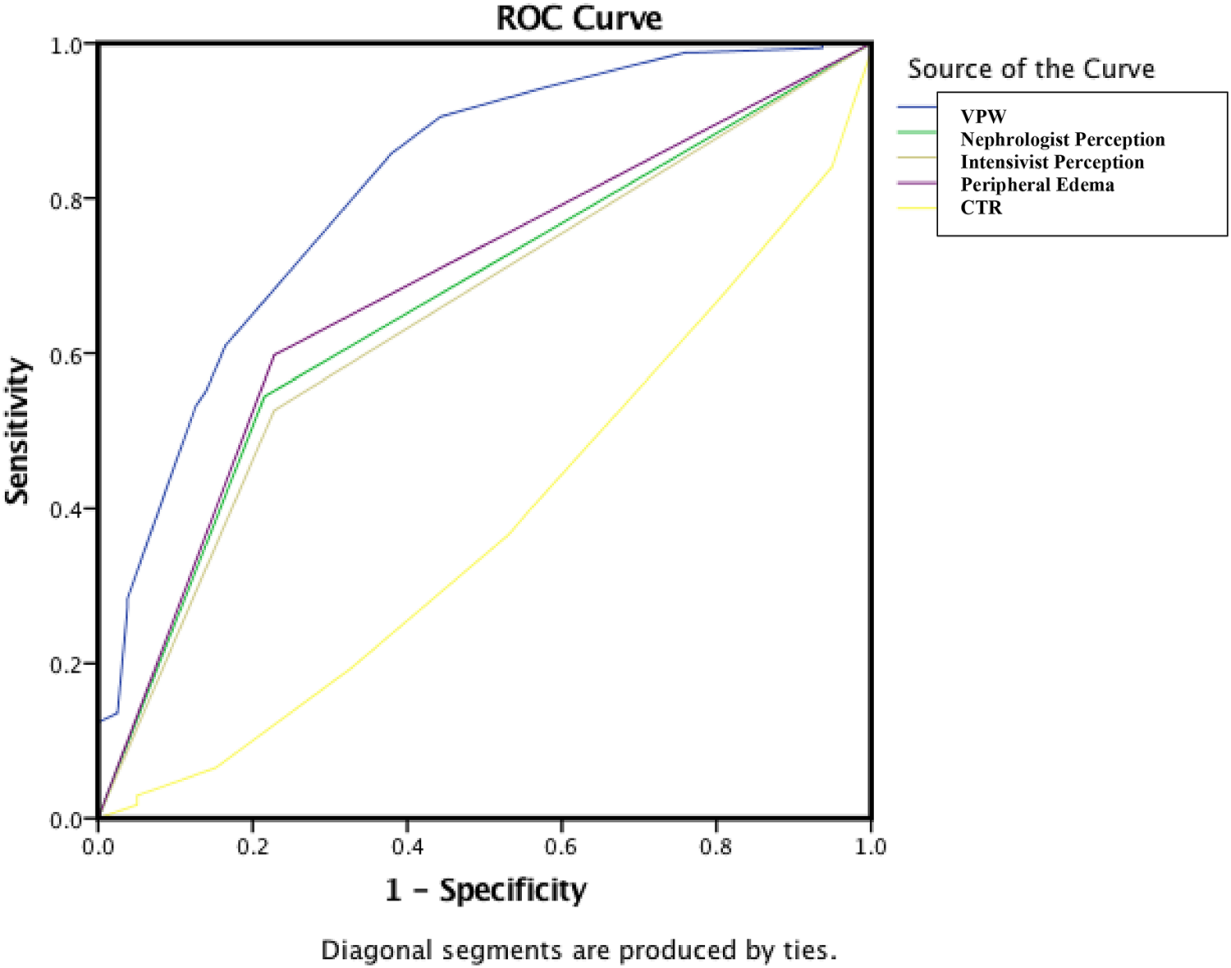
ROC curves of the VPW, clinician judgment, peripheral edema and CTR to predict the absence of hypotension during IHD.

ROC curves were generated to demonstrate the ability of the VPW, clinician judgment, peripheral edema and CTR to predict the absence of hypotension during IHD. ROC curve analysis yielded an AUC of 0.81 (95% CI 0.75 to 0.84), *P* < 0.0001, and the best accuracy was achieved with a VPW cutoff value of 70 mm (Fig. 1).

## Discussion

In this study, we evaluated predictive variables related to hypotension in critically ill AKI patients submitted to intermittent dialysis. We found that clinical judgment of patient fluid status using a systematic clinical approach, including patient history, symptoms, and physical examination, was not predictive of intradialytic hypotension. However, the diagnosis of sepsis, the use of norepinephrine, a lower MAP, a higher lactate level, the size of the VPW, the presence of peripheral edema and need for mechanical ventilation were strongly associated with hemodynamic tolerance of the dialytic procedure.

Critically ill patients with AKI requiring RRT are in a high-acuity, fast-paced and high-stakes environment in which critical thinking is imperative^16^. However, the physicians in charge of those critical decisions are not used to making the thinking process explicit (discussing cognitive biases, debiasing strategies and inductive reasoning). Most of the decisions are based on a more intuitive process of decision making^17,18^. Moreover, we found that overall agreement regarding the diagnosis of hypervolemia is poor among nephrologists and intensivists. In our study, we showed that the clinical judgment of hypervolemia does not predict hemodynamic tolerance of dialysis and that agreement about the presence of hypervolemia in individual patients was weak. Furthermore, the hemodynamic variables, perfusion parameters and other clinical data immediately before dialysis therapy that are usually used in the decision-making process based on the pattern recognition of a patient likely to become hypotensive during dialysis were not associated with hypotension during IDH.

The VPW, a measurement obtained from a CXR, is thought to be an indicator of the circulating blood volume^19,20^. There are clinical and statistical correlations between the VPW and volume overload in different critically ill patients, and the VPW can be used to evaluate the volume status of a patient regardless of the CXR technique used^21–25^. It has been reported that the measurement of the VPW may be useful for the estimation of body fluid volume status and that the VPW decreases significantly during dialytic procedures in patients undergoing hemodialysis or peritoneal dialysis^25,26^. In our study, we found that the VPW was strongly associated with the occurrence of hypotension during IHD, outperforming clinical judgment. Several limitations have been reported regarding VPW, such as effects of the variations in patient posture, radiographic technique, and ventilator-patient interactions that confront physicians. However, it should not deter the use of the VPW, but rather should fuel the need for the standardization of the interpretation of this. Of considerable to note, that all described tools to evaluate volume status, in critical care setting, have shown limitations and weakness^15^.

On the other hand, CXRs are normally available to most physicians and it is easily measured and has been shown to correlate well with invasive and noninvasive hemodynamic measurements. Despite several limitations, the portable CXR offers some utility in appraising volume status, although it is not to be relied on exclusively to volume responsiveness, should be evaluated as intravascular volume^15,20,21^.

Our study has several limitations. It is an observational study and, as such, even though we have tried to adjust for potential confounders, intradialytic hypotension is multifactorial and it is possible that other, unmeasured confounders, may impact on our results. As such, this study should be read as hypothesis-generating. Moreover, we did not compare the VPW to a direct measure of intravascular volume^27^. Although all the data were collected prospectively, many of the CXRs and dialysis procedures did not occur simultaneously. To minimize any potential bias this might have introduced, we limited our analysis to "matched" sets of measurements and CXRs obtained within three hours before the start of dialysis^28^. Additionally, there are current recommendations to limit the indications for CXRs to specific clinical contexts, such as changes in clinical status or the need for additional procedures^29^, and to use nondeleterious technologies, such as bedside ultrasound, to assess the patients^10,30^. Nevertheless, our data suggest that CXRs could still be used in resource-limited intensive care units that may not have access to bedside ultrasound and may be a useful tool in patients requiring RRT.

## Conclusions

In this observational study, clinical judgment of hypervolemia, as assessed by nephrologists and intensivists, did not predict hypotension during IHD. The predictive ability of the VPW can help physicians avoid inductive and deductive thinking and promote critical thinking.

## Supplementary Information

Supplementary Information.

## Data Availability

The datasets used and/or analyzed during the current study are available from the corresponding author on reasonable request.

## Abbreviations

RRT: Renal replacement therapy
IHD: Intermittent hemodialysis
VPW: Vascular pedicle width
AKI: Acute kidney injury
PAM: Mean arterial pressure
CXR: Chest X ray
CTR: Cardiothoracic ratio
ROC: Receiver operating characteristic
