# Supplementary material for: Prediction of hemodynamic tolerance of intermittent hemodialysis in critically ill patients: a cohort study
Source: Sci Rep. 2021 Dec 8;11:23610. doi: 10.1038/s41598-021-03110-4 (PMC8655072; doi:10.1038/s41598-021-03110-4)
Supplement: Supplementary file 1 — Supplementary Information. [file 41598_2021_3110_MOESM1_ESM.docx]

1. Flow Chart

**313 were ineligible**

313 meet exclusion criteria

- 118 requiring positive end-expiratory pressures higher than 5 cmH2O
- 110 chronic kidney disease
- 48 congestive heart failure
- 12 valvular heart disease
- 12 thoracic or cardiac surgery
- 8 pulmonary hypertension
- 5 pericardial disease

581

Patients were screened

248 underwent randomization

**20 met inclusion criteria, but excluded**

- 15 enrolled in other study
- 05 did not provide informed consent

268
